# Errors in Translating Medical Prescriptions

**Published:** 1846-08

**Authors:** 


*Errors in translating Medical Prescriptions.*—One of the most direct and palpable of the inconveniences arising from the pedantic custom of appending Latin directions to prescriptions, is the liability to errors of translation by the apothecary. Were the custom of employing Latin directions more general than it is in this country, mistakes from this source would be of every day occurrence ; for difficult as it would be for the majority of practitioners to use the learned tongue with invariable correctness, it would probably be much more difficult for apothecaries, in general, to interpret with accuracy for the patient. We do not deem it very derogatory when we confess that practical members of the profession generally have at their ready command "little Latin and less Greek," and the apothecaries will probably not consider it libelous to say that their available stock of dead languages is usually even less than this. Under such circumstances, amusing blunders are sometimes, as might be expected, committed. We meet with the following illustrations in the very interesting introductory lecture by Professor Dunglison, for 1845:

Chitty, in his "Medical Jurisprudence,' relates, that an action of slander arose between two medical practitioners,—the plaintiff, an apothecary—who, in England, is a sub-physician, having undergone a regular medical education—and the defendant, a physician,—which arose from the latter having prescribed some cathartic medicine for a nervous and costive old lady. The prescription, after directing the constituents of the medicine, added "*Repetatur si opus sit*," "Let it be repeated if necessary." The apothecary being absent, and his young man just from school—the latter, instead of construing the prescription in this manner, wrote on the label "to be repeated if it operates,"—translating "*si opus sit*," "if it operates." The consequence was, that the old lady, after having experienced the effects of the first dose, took another, and repeated it again and again, until she swooned from exhaustion. In alarm the physician was sent for, who incautiously exclaimed, and afterwards repeated to others:—"Tallman has killed my patient!" For this the action was brought; when forty shilings damages, and about two hundred pounds costs, were awarded to the plaintiff.

Another, a well known case, is given by Chamberlain in his "Tirocinium Medicum." A medicine was directed by a physician to to be given to a newly delivered female, and to be repeated *pro re rata*,—an expression constantly employed in prescriptions to signify, "as the thing may arise," or, in other words, "as occasion may require." The expression was not, however, familiar to a young compounder; who translated it *pro*, "for" *re*, "the thing," *nata*, "born;" or, as he wrote it, "for the little thing just born;" so that the infant took a dose which was intended for the parent."

